# Crop Pollen Development under Drought: From the Phenotype to the Mechanism

**DOI:** 10.3390/ijms20071550

**Published:** 2019-03-28

**Authors:** Jing Yu, Mengyuan Jiang, Changkui Guo

**Affiliations:** State Key Laboratory of Subtropical Silviculture, School of Agriculture and Food Sciences, Zhejiang Agriculture and Forestry University, Hangzhou 311300, China; yujing2009@zafu.edu.cn (J.Y.); jiangmengyuan1234567@126.com (M.J.)

**Keywords:** pollen development, drought, crop, mechanism, phenotype

## Abstract

Drought stress induced pollen sterility is a harmful factor that reduces crop yield worldwide. During the reproductive process, the meiotic stage and the mitotic stage in anthers are both highly vulnerable to water deficiency. Drought at these stages causes pollen sterility by affecting the nature and structure of the anthers, including the degeneration of some meiocytes, disorientated microspores, an expanded middle layer and abnormal vacuolizated tapeta. The homeostasis of the internal environment is imbalanced in drought-treated anthers, involving the decreases of gibberellic acid (GA) and auxin, and the increases of abscisic acid (ABA), jasmonic acid (JA) and reactive oxygen species (ROS). Changes in carbohydrate availability, metabolism and distribution may be involved in the effects of drought stress at the reproductive stages. Here, we summarize the molecular regulatory mechanism of crop pollen development under drought stresses. The meiosis-related genes, sugar transporter genes, GA and ABA pathway genes and ROS-related genes may be altered in their expression in anthers to repair the drought-induced injures. It could also be that some drought-responsive genes, mainly expressed in the anther, regulate the expression of anther-related genes to improve both drought tolerance and anther development. A deepened understanding of the molecular regulatory mechanism of pollen development under stress will be beneficial for breeding drought-tolerant crops with high and stable yield under drought conditions.

## 1. Introduction

Seeds and fruits originate from the reproductive process of flowering plants and are the main food source that sustains the survival of animals and humans [1]. Hence, producing enough crop yield is essential for population expansion and the biodiversity of animals and humans worldwide. However, plants are sessile organisms and cannot move like animals can when the environment is changing disastrously. They must face various types of abiotic and biotic stresses, such as drought, heat stress, salt stress, insect, pathogens and so on, which can restrict their productivity and even result in their death in extreme conditions [2,3].

Drought stress is considered to be one of the biggest threats to global crop yields [4]. The whole life cycle of crops is probably affected by drought, while the extent of damage, recovery capability and productivity change are closely associated with the developmental stage of plants suffering drought [5]. Medium water deficiency occurring at the juvenile stage only inhibits plant development and has almost no effect on the yield after they are re-watered. However, if crops encounter even trivial drought at the reproductive stage, their production is noticeably reduced and possibly insufficient, even if the rain returns, as the effect is irreversible [6]. Therefore, the reproductive stage is the most sensitive phase to drought. Unfortunately, exposure to water deficiency episodes often coincides with the reproductive phase of the plant life cycle [6].

The plant reproductive phase involves many processes, including floral initiation, anther and pollen development, pistil development, blossom, fertilization and seed development [6]. Of all the reproductive processes, the stage of anther and pollen developments is the most sensitive to drought [2]. Severe drought, which is already fatal for male development, is only able to limit female organ development, indicating that the female organs are insensitive to drought [6,7]. Therefore, it is crucial to understand the mechanisms and processes underlying drought-related male sterility for food security.

Pollen is formed inside the anther locules. Thus, the accurate determination of anther developmental stages is essential in anther and pollen studies under well-watered conditions. Until now, the morphological pollen development has previously been published for *Arabidopsis*, rice, wheat, *Brachypodium distachyon*, barley, etc. [8,9,10,11]. Generally, anther and pollen stages are closely related to the spike size and node number in rice, barley and wheat [12,13]. In this review, we selected rice as a model to illustrate its entire developmental program, including the 14 distinct morphological stages [9]. At stage 1, the L1, L2 and L3 layers of the floral meristem construct the anther primordium by cell division. Until stage 5, the anther structures with locule, wall, connective and vascular tissues are developed with the fast cell division and differentiation of the anther primordia. At stage 4, the sporogenous cells are generated. At stage 5, the epidermis, endothecium, middle layer and tapetum are formed. At stage 6, microspore mother cells (MMCs) can be found in the locule. Stage 7, stage 8 and stage 9 are the meiotic stages where the dyads and tetrads of haploid microspores are formed. At stage 8, the tapeta are secretory and begin to degrade. Following this, the free microspores are released (stage 9) and then vacuolated and rounded (stage 10). Through the first mitosis, a smaller generative cell and a vegetative cell with one vegetative nucleus are generated (stage 11). The generative cell is divided into two sperm cells and the mature pollen (stage 12). At stage 13 and 14, the pollen is mature and is released from the anther [9].

The definition of distinct developmental stages of anther in crops is crucial for understanding the fine stage that is easily affected by drought, and will be of help to improve crop yield under drought conditions. Here, we discuss the anther development under drought conditions from three perspectives including the phenotype, the physiology and the molecular mechanism.

## 2. Drought-Induced Injury to the Anther Structure

Drought stress occurring during the reproductive stage can significantly limit panicle development. Drought-treated spikelets are thin and pale with nearly white anthers and the discolored spikelets are desiccated rapidly after the panicle emerges [13]. Moreover, many sterile pollen grains with diluted cytoplasm and reduced starch are observed under water-deficient conditions [14]. To further explore the stage most sensitive to drought, anatomical methods including microscopic analysis and statistics are used to give us many new insights stimulated after drought treatment.

The nature of injury to the structure and function of anthers depends on the stage at which the plant experiences water stress. The transverse sections show that the pre-meiotic anthers have no obvious damage after medium drought treatment. However, during the meiotic stage, anther development has a visible difference between normal and drought conditions [2,13,15]. At stage 7, the defective anther locules and tapeta can be observed, as well as the abnormal meiotic cells under drought. At stage 8 and 9, the microspores fail to release, tapeta are vacuolated, and they even collapse under drought. The vacuolated tapeta block communications between microspores and outer cells. During the mitotic stage, some anthers from drought-treated plants contain disoriented microspores relative to the tapetum, which get dislodged from their normal peripheral location and increase the immature microspores, disintegrating the tapetum and expanding the middle layer (stage 11), which potentially squeezes the space between microspores. Thereafter, the fertile pollens are mature and filled with starch (stage 12), while the drought-treated pollens have little or no starch, and a hypertrophic endothecium and many degenerated microspores occur in the malformed anthers [2,13,15,16]. In short, the drought-induced abnormal phenotypes in crop anthers include defective anther locules, the degeneration of some meiocytcs, disorientated microspores, an expanded middle layer and abnormal vacuolizated tapeta.

Based on former studies, two stages of pollen development are more sensitive to drought: the meiotic and the mitotic stages. The meiotic stage of all cereals examined is known to be the most sensitive phase to drought; however, the mitotic stage is considered to be the most sensitive stage to drought in rice and wheat.

## 3. Physiological Bases for Abnormal Anthers under Drought

Drought affects pollen development by changing the homeostasis of the internal environment of plant cells, possibly by altering the intracellular sugars, hormones and reactive oxygen species (ROS) [2,17,18,19]. All these factors are interconnected to control the internal physiological homeostasis for survival in harsh environments.

### 3.1. Available Sugar and Reproductive Failure

Normal pollen grains accumulate starch for pollen germination and pollen tube growth, while sterile pollens have little or no starch [13]. Cytological evidences indicate that the starch is clustered in both connective cells and the endothecium layer of the anther, and in the transporting cells of lemma and palea, outside of anthers [13,20]. The ectopic accumulation of starch is associated with the reduced activity of invertase, the dominant enzyme of sucrose cleavage in anthers, and the changeless activities of starch synthase and ADP-glucose phosphorylase [20,21]. Reduced invertase activity would minimize the proper intake of sucrose, which harms the metabolic and developmental processes in anthers, resulting in pollen abortion [21]. Surprisingly, water deficiency during meiosis irreversibly jeopardizes invertase activity in anthers [21]. The loss in invertase activity results in an inability of the pollen to metabolize incoming sucrose to hexoses, which induces sterility. Moreover, drought limits photosynthesis in leaves and flowers, leading to the decreased supply and inconsistent distribution of soluble sugars to microspores, as well as the reduced synthesis of sugars in leaves [22]. However, the concentration of soluble sugars is usually shown to be higher in anthers under drought than under normal conditions. Possibly, the elevated soluble sugar only functions to regulate the osmotic pressure to combat drought. Meanwhile, the vacuolizated tapeta block the transfer and distribution of sugar to microspores, causing the defective sugar uptake of microspores [20,23]. Therefore, the changed carbohydrate metabolism and false accumulation are the main factors triggering drought-induced male sterility.

### 3.2. Role of Hormones in Drought-Induced Pollen Dysfunction

**Figure 1:**
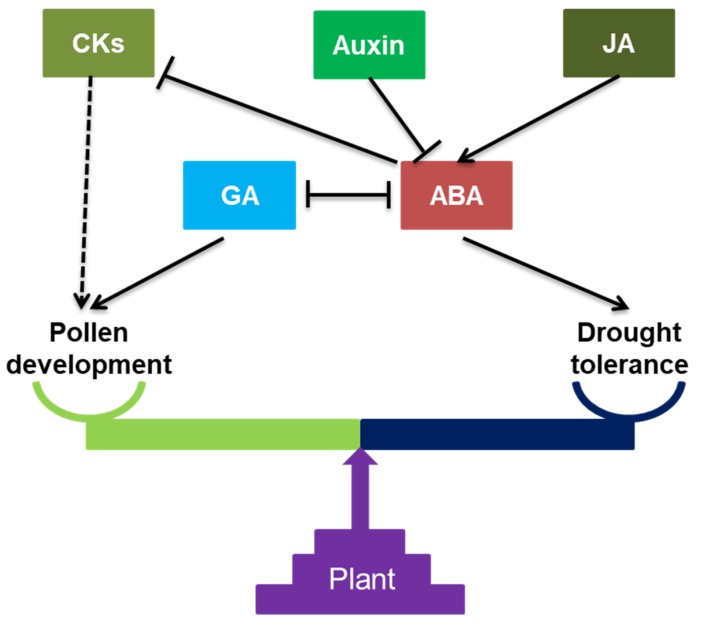
Interactions between hormones in regulating pollen development and drought tolerance. Dashed arrow: indirectly promote; solid arrow: directly promote; and T-bar: restrain.

Plant hormones, including gibberellic acid (GA), abscisic acid (ABA), auxin, jasmonic acid (JA) and cytokinin, are key regulators of reproductive development in plants. The balance of hormones is critical for pollen development and drought tolerance; the balance between pollen development and drought tolerance is regulated by the homeostasis of hormones (Figure 1). Under well-watered conditions, the ABA content in spikelets is very low [24], while low levels of GA cause sterility [25]. Drought induces ABA accumulation, which recovers a little after re-watering, and reduces the level of GA [16]. The ABA produced by leaves or other vegetative tissues is transported to spikes through long-distance phloem transport [17]. The elevated ABA increases the plant’s drought tolerance by increasing the osmotic pressure of anthers and it inhibits anther development; meanwhile, a moderate increase of GA under drought helps to improve pollen fertility. Loss of GA leads to the abnormal programmed cell death (PCD) of tapetal cells, and produces the aberrant ubisch body and exine [26]. Further studies indicate that reduced ABA content is required for PCD. Thus, the balance between ABA and GA is crucial for anther development, especially for timely tapetal PCD. Additionally, GA also plays a role in regulating levels of cell wall invertase and elevating sugar content in the stamen to maintain male fertility [27].

Drought decreases the content of indole-3-acetic acid (IAA), a species of auxin, resulting in the reduction of pollen ability and fertility of spikelets. Exogenous spray of IAA on plants during drought could preserve spikelet fertility [28]. Moreover, auxin significantly modulates sugar metabolites under drought [29]. Auxin concentrations could influence ABA synthesis and thus, like GA, the balance of auxin and ABA homeostasis is important for drought tolerance and anther development.

JA affects flower development and sterility in plants [30]. JA precursors are higher in the developmental stages of the reproductive organs in the sterile line than in the control [31]. Drought induces the increase of MeJA (methyl jasmonate) content in rice, which inhibits the number of stamens and fertility of pollen. The overproduced MeJA during drought stress stimulates the production of ABA to reduce grain yield [32]. Drought also induces cytokinin synthesis in plants, which coordinates the carbon and nitrogen metabolism to combat water deficit [33]. Cytokinin is a part of exine formation and pollen maturation [34], and the elevated content of cytokinin under drought may help to preserve anther development. Moreover, the level of cytokinin can be repressed by ABA to respond to drought [35]. However, its role in pollen development and drought remains poorly understood.

### 3.3. Overproduced ROS Disturbs Pollen Development

ROS acts as an important signaling molecule. ROS homeostasis is important as affable concentrations of ROS maintain normal growth and development in plants, while higher contents of ROS are toxic to plants. In rice, the timely production of ROS is associated with the initiation of tapetal PCD during anther development by the depletion of ATP (adenosine triphosphate) [36]. Drought induces the ROS level to disrupt ROS signaling to promote tapetal PCD earlier, resulting in pollen abortion. In the drought-resistant varieties, the antioxidant enzyme activities, including SOD (superoxide dismutase), POD (peroxidase), APX (ascorbate peroxidase), GR (glutathione reductase), and CAT (catalase), are increased during drought to remove the overproduced ROS to protect anther development [37]. However, the drought-sensitive varieties accumulate significantly more H_2_O_2_ and less H_2_O_2_-metabolizing enzymes such as CAT and APX under drought stresses [38]. Moreover, sugars, auxin, JA and ABA can regulate the redox state to integrate the ROS signaling pathway. Auxin can reduce the membrane lipid peroxidation and ROS accumulation, as well as affect underlying antioxidant enzyme activities in spikelets under drought stress [28]. Like auxin, ABA and JA induce antioxidant activity to eliminate the over-accumulated ROS during drought for organ protection [39]. The accumulation of sugars in stressed anthers reduces mitochondrial activity during the tricarboxylic acid cycle to eliminate the excessive production of ROS and the depletion of the ATP pool [40].

## 4. Molecular Mechanisms Regulating Anther Development under Drought

To study possible molecular mechanisms of regulating crop anther development under drought stress, microarray and RNA-seq methods are used to investigate the differentially expressed genes [13,41]. Microarray data from different sizes of spikelets in rice showed that a total of 1715 genes are differentially expressed under drought [13]. Of them, the meiotic and post-meiotic related genes are significantly suppressed, including *Defective Pollen Wall* (*DPW*), *Tapetum Degeneration Retardation* (*TDR*), *Cytochrome P450 Family Member* (*CYP704B2*), *Microspore and Tapetum Regulator 1* (*MTR1*) and *Wax Deficient Anther1* (*WDA1*) genes. These downregulated meiotic genes might interfere with the regulatory pathway, causing the meiotic process to inhibit normal pollen development which, to some extent, supports the idea that meiosis is the stage most sensitive to drought.

The sterile pollens contain little or no starch, which could be related to the reduction of carbohydrate supply and transport. The transcriptomic results show that genes encoding invertase (INV2), glycoside hydrolases, 6-phosphofructokinase and UDP (uridine diphosphate) -galactose/UDP-glucose transporters are downregulated in drought-treated rice anther [13]. Invertases function in hydrolyzing sucrose into glucose and fructose, and they are categorized into three subgroups including cell wall, cytoplasmic and vacuolar subgroups. Drought during meiosis selectively represses the expression of vacuolar isoform *IVR5* and cell wall isoform *IVR1*, but not the expression of *IVR3* in wheat [42]. In drought-sensitive varieties, the wheat anther cell wall invertase gene *IVR1* is significantly downregulated in drought-stressed anthers, while *IVR1* expression in drought-stressed SYN604 (drought-tolerant variety) anthers remains the same [23]. The variation in the expression of *IVR1* during drought in wheat could potentially be used to improve the drought performance of crops in the future.

Sucrose synthesized in leaves is transported to the anthers via long-distance phloem transport [43]. In rice anthers, *MST8* (monosaccharide transporter), *INV4* and *UGP1* (UTP-glucose pyrophosphorylase gene) expression is decreased by drought, supporting the sterility phenotype of the anther [44]. The expression of genes encoding sugar carriers and invertase shows that *OsCIN4* (cell wall acid invertase gene) and *OsHXK3* (hexokinase gene) is decreased in vacuolated microspores under drought, while the expression of *OsFK*I (fructokinase gene), *OsMST7* (monosaccharide transporter gene) and *OsSUT5* (sucrose transporter gene) is elevated in microspores and vacuolated microspores after drought treatment [40]. Moreover, *OsCIN4*, *OsSUT5* and *OsMST7* transcripts co-exist in the middle layer, tapetum and young microspores during the tetrad stage [40]. These results support the notion that the symplastic unloading of sugar occurs before the meiotic stage and the apoplastic transport of sugar occurs from the tetrad stage of anther development, suggesting that sugar transport during water deficit is critical. Trehalose-6-phosphate phosphatase (TPP) plays a role in catalyzing the dephosphorylation of the phosphosugar to form trehalose, which serves as sugar storage and metabolic regulator and protects against abiotic stress [45]. Overexpression of the rice *TPP1* gene in maize reduces the concentration of trehalose-6-phosphate (T6P), increases the concentration of sucrose in ear spikelets and improves maize yield under mild or severe drought conditions, possibly by enhancing the SnRK1 (sucrose non-fermenting-1-related protein kinase 1) activity [46].

Hormones and ROS play key roles in anther development. ABA synthetic genes, including *NCED* (9-cis-epoxycarotenoid dioxygenase) and *ZEP* (zeaxanthin epoxidase), are upregulated in rice spikelets [13], leading to a higher ABA concentration under drought. Drought induces ABA biosynthesis genes in anthers and ABA accumulation in spikes in drought-sensitive wheat varieties. Conversely, the ABA levels are reduced by reducing the expression of ABA biosynthesis genes and promoting the expression of the ABA degradation gene, *TaABA8′OH1* (ABA 8′-hydroxylase gene), in drought-tolerant wheat varieties [17]. The elevated ABA represses the expression of *TaIVR1* and further limits pollen development under drought. ABA acts antagonistically to GA [42], thus the GA signaling genes, including *LIPID TRANSPORTER*, *Defective Pollen Wall* (*DPW*), *CYP703A3*, *β-Ketoacyl Reductase* (*KAR*) and *MEIOTIC SERINE PROTEASE*, are significantly reduced after water deficiency [13]. The expression of *YUCCA* genes and their transcriptional regulators *SPL* (*SPOROCYTELESS*), *NGA* (*NGATHA*) and *TFL* (*TERMINAL FLOWER*) and auxin co-receptor genes, including *TIR1* (*TRANSPORT INHIBITOR RESPONSE1*), *IAA* (*INDOLE-3-ACETIC ACID*), *ARFs* (*AUXIN RESPONSE FACTORS*) and *PINs* (*PIN FORMED*), is suppressed by drought in rice spikelets [28]. In addition, the protein disulfide oxidoreductase activity and cell redox homeostasis-related genes, such as CC-type *GRXs* (glutaredoxins containing a CC[M/L][C/S] active site), are also altered in the anther to antagonize the overproduced ROS by drought to protect the disrupted anther lobe differentiation and disabled meiotic entry of sporogenous cell progenies [41]. During meiosis, some differentially expressed genes, encoding β-carotene hydroxylase and cytochrome P450 monooxygenase, probably protect against oxidative damage induced by drought [47]. In wheat, a *TaOPR2* gene (12-oxo-phytodienoic acid reductase), induced by drought and involved in the biosynthesis of JA, is isolated from the thermo-sensitive genic male sterile wheat cultivar BS366. Overexpression of *TaOPR2* can preserve the male sterility phenotype of *Arabidopsis* mutant *opr3* [48]. The differentially expressed histone *H2A* and dehydrin *DHN1* may prevent chromatin stabilization and dehydration under drought [47]. Furthermore, many novel miRNAs and stress-regulated miRNAs that may function in stress response in rice inflorescences are identified [49].

To explain the difference in rice spikelet fertility between the drought-sensitive genotype ‘IR64′ and the drought-tolerant genotype ‘Moroberekan’, the drought-responsive and developmental changes in protein abundance were examined using two-dimensional (2-D) gel electrophoresis and MALDI-TOF MS (Matrix-Assisted Laser Desorption/Ionization Time of Flight Mass Spectrometry). The results show that the recovery capability of the drought-tolerant rice genotype is higher at the anther proteomic level than that of the drought-sensitive genotype [5]. A total of 12 proteins, absent from the drought-stressed pollen proteome, were identified, such as secretory class III peroxidase 28, cysteine proteinase EP-B1, cytosolic ascorbate peroxidase and so on. Simultaneously, eight newly drought-induced proteins were identified, including a glyceraldehyde-3-phosphate dehydrogenase, three beta-expansins, a pectinesterase inhibitor domain containing protein, an actin binding protein (ADF5), etc. [5]. From the spikelets of the rice drought-tolerant genotype ‘N22’, 11 differentially expressed proteins under water deficiency were separated, such as pollen allergens, low-molecular-weight heat shock proteins (HSPs), beta expansin, soluble inorganic pyrophosphatase and so on [50]. The identified proteins will be further studied for their function in drought stress during the reproductive stage. These genotypes may be ideal candidates for developing drought-tolerant rice.

Using the transgenic method, a wealth of genes which function in improving crop drought tolerance in the reproductive stage were identified. Overexpression of the rice *MID1* (*MYB Important for Drought Response1*) gene, induced by drought and expressed in the tapetum, decreases the ratio of deformed anther locules, abnormal tapetum, degenerated microspores and an expanded middle layer, possibly by directly regulating *KAR*, *Hsp17.0* and *CYP707A5* to improve drought tolerance and anther development [2]. Overexpression of *OsDIL* (*Oryza sativa Drought-Induced LTP*), mainly expressed in anthers, alleviates genes required for drought-suppressed anther development, including *OsC4*, *CYP704B2* and *OsCP1* (cysteine protease gene), to maintain normal pollen development at the time of water deficiency [15]. Overexpression of *OsAHL1* (AT-hook content nuclear localized protein) significantly improved drought resistance at the panicle development stage in rice by elevating the activity of POD and relieving the degradation of chlorophyll [51].

**Figure 2:**
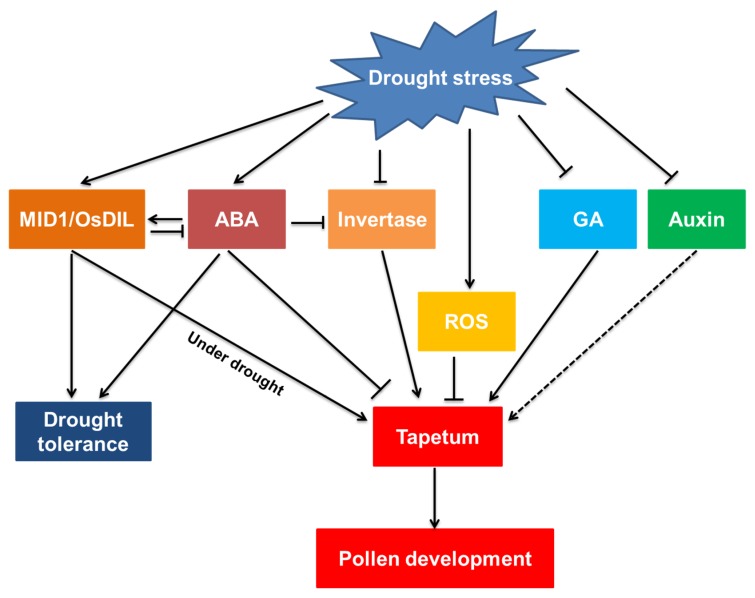
Model of the effect of drought stress on pollen development. Dashed arrow: indirectly promote; solid arrow: directly promote; and T-bar: restrain.

Overall, both the meiotic and mitotic stages are sensitive to drought. Drought-induced tapetal dysfunction is the main cause of pollen abortion by affecting the distribution of soluble sugars to microspores. Drought inhibits the concentrations of GA and auxin as well as the supply of sugar, and promotes the levels of ABA and ROS in anthers for suppressing anther development, leading to male sterility. Furthermore, the expression of genes required for anther development, hormone metabolism genes, ROS-scavenging genes and their signaling genes, are all altered to regulate anther development during drought (Figure 2).

## 5. Perspectives

Previous effects have revealed that many drought-responsive genes, identified by RNA-seq and proteomics, are related to anther development under drought. Some of them have had their roles in anther development during water deficiency functionally confirmed. However, our understanding of anther development under drought is just beginning.

First, the function of most drought-induced or -suppressed genes remains unclear. Hundreds of drought-response genes in anthers have been identified, while their expression pattern, cellular or tissue localization, interactions and functions have not yet been reported. Therefore, the gain and loss functions of these genes need to be developed to further study their functions in anthers under drought. Furthermore, the use of a gene editing approach to edit these functional genes for molecular breeding would also be beneficial.

Second, how to control hormones and sugar to achieve higher fertility under drought is still poorly understood. Drought limits the concentration of GA and sugar in the anther. Whether spraying exogenous GA or sugar on plants during drought can preserve the pollen fertility still remains unknown, as does the question of how the signal transduction is transferred. Therefore, an exogenous spraying experiment coupled with a transcriptomic analysis might help us answer these questions.

Third, invertase activities are essential for anther development. However, how these activities function remains largely unknown. Exploration of their molecular mechanism by co-expression and interaction analysis at the reproductive stage under drought might help to unveil this mystery.

Finally, drought usually occurs at the reproductive stage. We can change the lifecycle of a crop by molecular methods or by field management to avoid drought stress at the reproductive stage.
